# On Wounds and Injuries of the Abdomen and the Pelvis

**Published:** 1847-07-01

**Authors:** 


### On Wounds and Injuries of the Abdomen and the Pelvis. Being the Second Part of the Lectures on some of the more important Points in Surgery. By *G. J. Guthrie,* F.R.S., &c. 8vo, pp. 72. Churchill, 1847.

In fact this second portion of the work we had a short time since occasion to notice so favourably, has never appeared in the form of "Lectures," or enjoyed the very questionable advantage of being interred amidst the masses of unreadable matter, which fill up the overgrown columns of our weekly cotemporaries. We much regret, however, to find that the non-delivery of the lectures was occasioned by the occurrence of a domestic calamity, which temporarily unhinged the robust mental constitution of our veteran surgeon. Happily he was possessed of ample materials for the illustration of the subject he had contemplated lecturing upon, and the arranging these for publication served at once to beguile away his Winter evenings—(how much is expressed in these few words those who have suffered from bereavements alone can tell)—and to present another useful work to the medical public. Like its predecessor, it chiefly consists of succinct accounts of cases which came under the author's observation during his campaigns, with subjoined commentaries and practical deductions. He has however availed himself likewise of the labours of other writers upon military surgery, so that his work presents us with a brief epitome of what is known upon the subject. We prefer presenting the general conclusions, with which he terminates it, to our readers' notice, before offering any comments upon any of them. They are as follow :—

"1. Severe blows on the abdomen give rise to the absorption of the muscular structures, and the formation, in many instances, of ventral hernia; which may in some measure be prevented, during the treatment, by quietude, by the local abstraction of blood, and by the early use of retaining bandages. 2. Abscesses in the muscular walls of the abdomen, from whatever cause they arise, should be opened early; for, although the peritoneum is essentially strong by its outer surface, it is but a thin membrane, and should be aided surgically as much as possible. 3. Severe blows, attended by general concussion, frequently give rise to rupture of the solid viscera, such as the liver and spleen, causing death by hæmorrhage. When the hollow viscera are ruptured, such as the intestines or the bladder, death arises from inflammation. 4. Incised wounds of the wall of the abdomen of any extent rarely unite so perfectly (except perhaps in the linea alba) as not to give rise to ventral protrusions of a greater or less extent. 5. As the muscular parts rarely unite in the first instance after being divided, sutures should never be introduced into these structures. 6. Muscular parts are to be brought into apposition, and so retained principally by position, aided by a continuous suture through the integuments only, together with long strips of adhesive plaster, moderate compression, and sometimes a retaining bandage. 7. Sutures should never be inserted through the whole wall of the abdomen, and their use in muscular parts, under any circumstances, is forbidden; unless the wound, from its very great extent, cannot be otherwise sufficiently approximated to restrain the protrusion of the contents of the cavity, the occurrence of which case may be doubted. 8. Purgatives should be eschewed in the early part of the treatment of penetrating wounds of the abdomen. Enemata are to be preferred. 9. The omentum, when protruded, is to be returned, by enlarging the wound, through its aponeurotic parts if necessary, but not through the peritoneum, in preference to allowing it to remain protruded, or to be cut off. 10. A punctured intestine requires no immediate treatment. An intestine, when incised to an extent exceeding the third of an inch, should be sewn up by the continuous suture. 11. The position of the patient should be inclined towards the wounded side, to allow of the omentum or intestine being closely applied to the cut edges of the peritoneum. Absolute rest, without the slightest motion, should be observed. Food and drink should be restricted, when not entirely forbidden. 12. If the belly swells, and the propriety of allowing extravasated or effused matters to be evacuated, seems to be manifest, the continuous suture or stitches should be cut across to a certain extent, for the purpose of giving this relief. 13. If the punctured or incised wound is small, and the extravasation or effusion within the cavity seems to be great, the wound should be carefully enlarged, and the offending matter evacuated. 14. A wound should not be closed until it has ceased to bleed, or until the bleeding vessel has been secured, if it be possible to do it. When it is not possible so to do, the wound should be closed, and the result awaited. 15. A gun-shot wound penetrating the cavity can never unite, and must suppurate. If a wounded intestine can be seen or felt, its torn edges may be cut off, and the clean surfaces united by suture. If the wound can neither be seen nor felt, it will be sufficient for the moment to provide for the free discharge of any extravasated or effused matters, which may require removal. 16. A dilatation or enlargement of a wound in the abdomen should never take place, unless in connexion with something within the cavity rendering it necessary. 17. When balls lodge in the bones of the pelvis, they should be carefully sought for and removed, if it can be done with safety and propriety. 18. In a wound of the bladder, an elastic gum catheter should be kept in it, until the wound is presumed to be healed—unless its presence should prove injurious from excess of irritation, not removeable by allowing the urine to pass through it by drops as it is brought into the bladder. 19. In all cases in which a catheter cannot be introduced, in consequence of the back part of the urethra or the neck of the bladder being injured, an opening for the discharge of the urine should be made in the perineum. 20. The treatment of all these injuries must be eminently antiphlogistic, principally depending upon general and local blood-letting, absolute rest, the greatest possible abstinence from food, and in some cases from drink, the frequent administration of enemata, and the early exhibition of mercury and opium, in the different ways usually recommended, with reference to the part injured." P. 72.

*Ventral Hernia.*—This is the never-failing consequence of a musket-ball penetrating the walls of the abdomen, and it is also usually attendant upon the division of the parietes by a sharp cutting instrument. Moreover, the same result may follow a severe *bruise* without any rupture of muscular fibres being perceptible. Of this Mr. Guthrie relates two cases. In the one, a large flat piece of shell struck the left iliac region, producing a severe and painful bruise, the whole of that side of the belly becoming first black and then discoloured. The patient recovered with little or no treatment; but, upon examining the part some months after, Mr. Guthrie found that the whole of the muscular portion of the wall corresponding to the seat of injury had been removed by absorption, the tendinous portions having also become very thin, protruded on any effort being made, so as to constitute a circular broad-based ventral hernia, requiring the application of a bandage. In the other case, the belly was struck by the swinging round of the spanker-boom of a small vessel in the Tagus, and the same absorption of muscle and formation of hernia resulted. Mr. Guthrie suggests that, perhaps a more active treatment of the accident at first, and the earlier application of a bandage, might possibly have prevented this consequence.

The numerous operations of late performed for the removal of ovarian tumours have shown that, when an incision, even of great extent, has been made along the course of the *linea alba,* the union of this part may be sufficiently firm to resist any hernial protrusion. Mr. Walne sent a woman for Mr. Guthrie's inspection, in whom he had made an incision 14 inches long, and yet at the end of as many months all was firm. In other cases, where adhesion had been prevented by the ligature from the peduncle of the tumour passing out between the edges of the wound, Mr. W. has found protrusion. So exact may the union of the divided peritoneum become, that a minute inspection after death has been required to detect the site of the incision. Dr. F. Bird states that, of 12 cases in which he had opened the abdomen, recently examined by him, the incision in five did not exceed three inches ; in five it reached from $4\frac{1}{2}$ to 5 inches ; in one to 8 inches ; and in one to rather less than 12. With the exception of one case the incisions were made along the *linea alba,* without involving the muscular structure. Soon after the wounds healed in general, a very marked contraction of the abdominal walls took place ; so that an incision of five inches mostly became replaced by a cicatrix of scarcely more than an inch. Whenever such contraction occurred, no tendency to hernia manifested itself ; while in two cases, in which the incisions were from 8 to 12 inches long, little contraction followed, and protrusion of the cicatrix, or a hernia, ensued. In one case, in which a small incision was made transversely into the outer edge of the rectus and internal portion of the obliquus externus, hernia quickly followed. In all the cases the same mode of closing the wound was observed, viz. by the introduction of two interrupted sutures in every inch of incision, the skin alone being included.

"If it should be proved that incisions may be made with more confidence of success than hitherto has been supposed, and as these sections may lead us to believe, it will only confirm what I have constantly repeated from year to year in my lectures, that penetrating wounds of the abdomen, without injury of the viscera, when properly treated, are not so dangerous as they were generally supposed to be. In the sections made by other surgeons for the removal of diseased ovaries, ligatures have been sometimes used for the purpose of bringing the divided muscular and tendinous parts together, and although I forbid their employment as a general rule, I do not wish to imply that in very extensive transverse wounds they can be entirely dispensed with, if only to prevent the immediate protrusions which the suture through the skin might not be equal to resist in every case." P. 10.

*Employment of Sutures.*—The latter portion of the above extract leads us to Mr. Guthrie's views upon the mode in which sutures are most advantageously employed. His multiplied experience during the war, confirmed by what he observed afterwards in cases of ligature of the iliacs, having convinced him that the walls of the abdomen, formed of mingled muscular and tendinous structures, cannot be brought to unite permanently, save by the intervention of cellular texture, he disapproves of the endeavour to keep these parts in contact by means of sutures, which are of no permanent service, and may create much present irritation. Chelius, Graefe, Weber, and South, seem however to approve of this stitching the muscles together, although Chelius gives also directions for their immediate removal, if dangerous symptoms, such as inflammation, vomiting, hiccough, &c. supervene; but Mr. Guthrie believes that such accidents are much better obviated by avoiding the practice, which English surgeons have long discarded, as relates to other portions of the body. He brings the edges of the wound together by means of a small needle and fine silk, passing this only through the skin and contiguous cellular tissue, and employing the "continuous suture without puckering, precisely in the manner a tailor would fine-draw a hole in a coat or a lady a cut in a cambric handkerchief." This gives a certain degree of support to the parts beneath, which is also to be further afforded by strips of plaister extending for some distance around the body, a bandage being rarely of use, while, if it causes pressure, it will be mischievous. Great attention should be paid to the position of the patient inclining the body towards the wound, so as to keep the intestinal or omental peritoneum, if possible, in contact with the divided portion, until it has adhered to this. In such position he must be rigorously maintained without any change, until all hope of adhesion occurring has passed away.

*Management of the Protruded Parts.*—When omentum is the protruded part, the rule usually laid down in surgical works is to return it into the cavity of the abdomen again, and endeavour to retain it there by the application of sutures as near the peritoneum as possible. To this Mr. Guthrie demurs, believing that the best practice consists in retaining this substance between the cut edges of the peritoneum, without any strangulation or even compression however, its adhesion there forming the best barrier against the extension of inflammation. He believes, too, that the advice given to enlarge the opening into the cavity when this is insufficient for the return of the omentum is bad, inasmuch as the obstacle to its reduction is not formed by the peritoneum but by the abdominal aponeuroses, the slightest division of which will almost always allow of the easy return of the omentum. Small wounds in the peritoneum, when covered over with healthy parts, are not usually followed by bad consequences, but the most disastrous ones may attend a large one ; so that the greatest care should be taken not to augment the size of one already existing. If the omentum is adherent, inflamed, torn, jagged, or in a state of suppuration or gangrene, it should not be returned, nor should it be surrounded by a ligature, or cut off; but left to itself and treated in the most simple manner. It will gradually retract and become withdrawn into the cavity of the abdomen. Observation of several cases of neglected penetrating abdominal wounds during the Peninsular war, convinced Mr. Guthrie of the propriety of thus leaving the omentum between the edges of the wounded peritoneum, closing the integuments over it by the continuous suture. Larrey recommends leaving the omentum which cannot be returned *in situ*, and relates cases shewing the power which nature exerts in drawing it gradually into the abdomen. Mr. Guthrie has also seen such, but he has also seen fatal consequences follow both the leaving the part outside and the cutting it off. If, indeed, it be very much bruised or injured it may be cut off, but, as the general practice, he prefers returning it as far as the edge of the peritoneum, and bringing the skin over this.

The *intestine*, in any condition short of that of mortification, should be returned to the abdomen, but the directions given for this purpose by Chelius are objected to by Mr. Guthrie. These are, that the opening of the peritoneum should be enlarged when the reduction is difficult, the finger introduced to feel that the gut has not passed between the muscular interspaces, and that the patient should be so placed as to prevent the intestines pressing against the wound. The two first of these are unnecessary and mischievous, and the last erroneous, since the very best thing that could happen to consolidate the union would be to bring the intestine in contact with the divided peritoneum. When the protruded intestine is wounded, if it be a mere puncture or a very small cut, the bowel may be returned just the same, the pressure of the containing parts preventing effusion, the development of inflammation being closely watched. Wounds, however, sometimes of apparently the slightest appearance, give rise to terrible consequences, while others proceed favourably, in spite of every aggravating circumstance.

*Wounds of the Intestine.*—In his second and third "Lectures," Mr. Guthrie furnishes us with an elaborate view of this subject derived from his own observations, and those of preceding writers. After describing the experiments of Travers and Gross, he observes upon these latter :—

"The important conclusions to be deduced from his experiments, 1, that wounds not exceeding four lines in length, no matter what their direction may be, are not so apt, as might be supposed, if left to themselves, to be succeeded by extravasation of the contents of the intestinal tube; and that, in the majority of cases, Nature, properly aided by art, is fully competent to effect reparation. 2. That wounds of the bowels, to the extent of six lines, whether transverse, oblique, or longitudinal, are almost always, if not invariably, followed by the escape of the contents of the bowel, and the consequent development of fatal peritonitis. It may, therefore, be concluded, from these experiments made on animals, as far as they can be relied upon in reference to man, that every wound of the bowel, of such extent as shall not admit of its being temporarily filled up by the protrusion and eversion of its internal or mucous coat, ought, if possible, to receive assistance from art, which can only be given with advantage in the first instance." P. 18.

That this last conclusion, founded as it may easily be upon experience in the human subject, is a correct one, may at once be admitted; and, indeed, experiments upon the lower animals might induce a fallacious security, since Nature seems in so many instances to have endowed them with the power of resisting an extent of lesion to which man succumbs; and as, in them, the third tunic of the intestines, (the fibrous lamella of Cruveilhier, and the membrana nervosa of Haller), the most important of the tunics, that in which hypertrophy has its seat and in which adhesion after division readily takes place, varies much in strength and elasticity in different animals. Mr. Quekett has shown that it is most developed in reptiles, and more so in the carnivora than in man and the herbivora.

After describing the various ingenious modes by which surgeons have endeavoured to maintain the divided edges of the intestines in juxta position, Mr. Guthrie quotes the following observations from Dr. Gross' "Critical Enquiry into the Nature and Treatment of Wounds of the Intestines."

"Of the four methods, that of introducing the suture through the cellular fibrous lamella is the least objectionable, as it enables us to bring the serous surfaces into more accurate apposition. When the needle is conveyed through all the tunics, there must necessarily be some degree of puckering, whereby the mucous lining will be forced between the lips of the wound, if not beyond the level of the peritoneal membrane. By such an arrangement, the adhesive process would be retarded, and if the stitches were to lose their hold, or if the bowel should not become glued to the neighbouring parts, fæcal effusion might occur, followed by its whole train of evil consequences.

"In making the continued suture, I would, therefore, recommend, that the needle be carried through the cellular fibrous lamella, or between the muscular and mucous membrane, and not across all the tunics, as is generally advised by authors. The lips of the wound should be held parallel with each other, and the stitches, drawn with considerable firmness, should not be more than one-twelfth or one-eigth of an inch apart. The needle is to be introduced a short distance, say half-a-line, from the peritoneal edge of the opening, and brought out at the corresponding point at the opposite side. The first stitch should be one line from one angle of the wound, and the last about the same distance from the other, care being taken to secure each with a double knot, and to cut off the extremities of the suture close to the surface of the tube. The instrument which I prefer, and which I employed in nearly all my experiments, is a long slender sewing needle, armed with a waxed, but strong and delicate, silk thread. The operation should be performed as expeditiously as is consistent with safety, and the bowel handled in the gentlest manner possible." P. 26.

These conclusions have only their foundation upon experiments upon animals, and Mr. Guthrie at present demurs to their practical adoption.

"The continuous sutures of Bertrandi, of Travers, of Dupuytren, of Lembert, of Jobert, of Gross, and of Nuncianti, appear to resemble each other so much as to be essentially alike; the object of all being to close the bowel effectually. The later writers have attended more than their predecessors, to turning the cut edges of the intestine inwards, so as to bring the serous surfaces external to the cut edges in contact, when the ligatures are duly tightened—an important addition to the operative process. It remains to be proved, from observations and from trials made on man, whether the mode of proceeding which refrains from passing the needle and thread through the inner or mucous membrane, or that which effects this object, is the best. I am of opinion that the continued suture through all the coats of the intestine is the best; and shall continue to think so until it shall be proved that the ligature passes into the bowel in both methods; a result which is doubted, when the inner membrane remains intact." P. 26.

A punctured wound of the intestine, or one not more than a third of an inch in length does not call for interference; and even when the external wound is large we must not make it still larger for the purpose of investigating the condition of the intestines, unless any portion of these protrude, their contents are discharged, or hæmorrhage occurs. If, however, in the course of two or three hours' time we find the belly swelling from effusion or extravasation of blood, prompt interference gives our patient the only chance. The external wound must then be enlarged, the effused matters sponged up, and the bowel or artery secured by suture. So, too, we may have to re-open and even enlarge a wound which has already been closed by suture over a wounded intestine, providing swelling and ungovernable inflammation indicate this procedure. If, when we are enabled to examine the wound of the intestine, we find this to be very small, but not filled up by the mucous membrane of the gut, "a tenaculum may be pushed through both cut edges, and a small silk ligature passed around, below the tenaculum, so as to include the opening in a circle, a mode of proceeding I have adopted from analogy, with success, in the external jugular vein, without impairing its continuity." Or one, two, or more continuous stitches may be made with a very fine needle and silk, cut off in either case close to the bowel. If the person survive, the threads or sutures will be carried into the canal. When the intestine is largely injured, longitudinally or transversely, or is more or less completely divided, the continuous suture, as already stated, is to be preferred to all others. When, after wounds of the abdomen, considerable hæmorrhage occurs, the wounded vessel must be sought for. "If the blood comes from one of the mesenteric arteries, or the epigastric, the wound is to be enlarged, until the bleeding artery is exposed, when ligatures are to be placed on its divided ends, if they both bleed. I have seen this vessel tied several times with success."

There is a much greater degree of danger attendant on wounds of the small intestines, than of the large ones; that is, recoveries more frequently follow wounds of the colon in its various parts, and of the rectum, than of the jejunum or ileum.

In his third Lecture, Mr. Guthrie relates several examples of recovery after the most serious injuries by musket-balls penetrating the abdomen; and, alluding to the dictum of the late Dr. Thomson, founded on what he had seen after Waterloo, that the less the operations of Nature are interfered with the better the chance of recovery, properly observes that this is correct only in the absence of clear indications for interference. "When they are present, the do-nothing system is generally followed by death. A well-regulated interference is likely to be more successful."

*Effusion of Blood into the Abdomen.*—Petit the younger first pointed out that extravasations of blood were not diffused over and between the entire surfaces of the intestines, provided the person outlived the period of extravasation; and that general effusion only took place shortly before or immediately after death. When blood is effused in only moderate quantities, and is circumscribed by the compression of the abdominal parietes, it may be evacuated by the wound if this be sufficiently open, and the patient may recover if the accompanying inflammation is not propagated along the peritoneum, or becomes subdued. If its quantity be very small it may become absorbed; but where this is not the case it may then became a source of irritation. Petit and Larrey showed that it is then soon surrounded by fibrin, and thus cut off from the general cavity—a pouch or *foyer*, restrained within certain bounds, being thus formed. It now, however, ceases to be bland and harmless, and may, like any other foreign body, excite inflammation and suppuration. This occurs at a later period only, at the 10th, 12th, or a later day, after perhaps the original inflammatory symptoms have all subsided. With the general symptoms, pain and swelling near the wound manifest themselves, and sometimes fluctuation may be at once distinguished. If an incision be deemed advisable, it will be better first to pass down an exploring needle or a very fine trochar, and if this indicates that a bloody, purulent, or other fluid, is distending the abdomen, the original wound should be enlarged, or a more dependent opening made at once.

The formation of these pouches, reservoirs or *foyers* are, however, so rare in the natives of our northern climate, that Mr. Guthrie looks upon them as very exceptional occurrences.

*Artificial Anus.*—Mr. Guthrie enumerates some of the various procedures which have been devised for the relief of this disgusting infirmity, but adds nothing from the stores of his own experience. In truth, as he remarks, this portion of surgery has been almost exclusively cultivated on the continent, where the infirmity is far more prevalent than in this country. Whether the reason of this he assigns, namely, that the "hardy natives of Britain and Ireland seldom outlive the processes ending in mortification," is the correct one, we know not; but their seldomer outliving these, appears to us, at the least, a singular proof of their greater hardiness.

*Wounds of the other Viscera.*—We can only cursorily glance at Mr. Guthrie's numerous observations upon these. Wounds of the *Liver* are very serious, though not necessarily fatal; a few persons recovering altogether, some imperfectly, and more dying either during the immediate or the secondary inflammatory action which is set up. Pain in the right shoulder and cramps of the muscles of the arms are common symptoms, joined to those of ordinary peritonitis. Several cases of recovery after severe wounds, giving rise to the discharge of blood and bilious coloured fluids, are related; but all the cases in which the *gall-bladder* was wounded proved fatal. Wounds of the *Stomach* are generally fatal, except when they are confined to its upper and anterior part, in which case the patient may survive. If the wound is small, it must be treated by the continuous suture, just as that of the intestines ; and if the external aperture through which its contents are effused is too small to admit of the wound in the organ being sown up, it should be enlarged. This is, however, only a suggestion for the prevention of otherwise a certain death, Mr. Guthrie never having put it into force yet. In cases of wounds of this organ, the greatest caution in allowing food, especially of a solid character, must be long observed. In allusion to a case of idiopathically inflamed stomach, not marked by pathognomic symptoms, and in which suppuration had converted the coats of the organ into the appearance of a honey-comb, Mr. Guthrie makes the following general observation.

"If there be a symptom more generally observable than another in all cases of dangerous wounds of the abdomen, it is *anxiety*—not only of the mind, as shewn by the countenance in a very expressive manner, but of the body, as demonstrated by the great uneasiness. In some cases it is a more certain sign than the pulse of great mischief; in others, more distinctive than pain, which is sometimes referred to a part unaffected ; and is in all indicative of the necessity of a corresponding attention on the part of the practitioner; for, while it is present, although other symptoms be mild, the patient is in imminent danger." P. 55.

Cases of fistulous openings into the stomach are by no means rare. Dr. Watson has collected, in the *American Journal of Medical Sciences*, for 1844, many instances of such, and of incisions made into the organ accidentally or for the removal of knives, &c. "Ploucquet, in his *Bibliotheca*, has enumerated nearly a hundred writers under the head '*Pantaphagus*,' who have related such cases, and as many more under the heads 'Vulnus' and 'Ventriculus,' and Hevin, in the 1st. vol. of the *Memoires de l'Academie de Chirurgie*, has related some of the most interesting cases of those who have swallowed knives, &c. up to his time." Incised wounds of the stomach were rarely met with during the Peninsular war. Gun-shot wounds were not unfrequent, and so fatal that, while Baron Percy estimates the successful cases as 4 or 5 in 20, Mr. Guthrie only calculates one-half of these.

Wounds or rupture of the *Spleen* are usually fatal, from the abundant hæmorrhage they give rise to. Mr. Guthrie, during the Peninsular war, heard of no case, in which the spleen protruded through a wound, which recovered ; although instances are related by authors in which this organ was successfully removed after injury. Wounds from bayonets, swords, &c., are not always fatal, since cicatrices are sometimes found in the organ corresponding to the external wound. Wounds of the *Kidney* are not so fatal as those of the spleen, although the complications they eventually give rise to render them scarcely less dangerous. Owing to the lingering character of some of the results, these are often unknown, the patient being lost sight of. The opinion once entertained, that wounds of the *bladder* are necessarily fatal, is now known to be erroneous ; and when this organ is wounded below, or where it is not covered by peritoneum, patients recover often almost unaided. Dr. Thomson saw fourteen cases recovering at Brussels after Waterloo, in which the bladder had been penetrated by musket-balls. Mr. Guthrie has never seen a case recover after the urine has found its way into the general cavity of the abdomen, the patient generally dying in from three to six days.

*Treatment of Injuries of the Abdomen.*—Mr. Guthrie protests against the practice once in vogue of bleeding a patient immediately after the injury, thus delaying the necessary reaction, and preventing the agglutinative process which may prevent inflammation extending to the peritoneum. The abstraction of blood must entirely be guided by the existence of inflammation; and although, on the occurrence of this, very large quantities may have to be taken away, he is certain that he has seen cases in which depletion has been injurious from excess, the marks of intense inflammation after death having been wanting. However, in various parts of his work, he shows that he is an advocate for a very free use of the lancet, the employment of entire abstinence, and the other usual means of subduing violent inflammatory action, which is the character traumatic peritonitis, occurring in robust subjects, usually puts on. Indeed the idea, during our perusal of the cases, has several times occurred to us, that the subsequent reparative power was, in all probability, impaired in many of these, by the energy of the prior proceedings, and that the substitution of large doses of opium, after at least the first bleeding, and the permission of a somewhat better diet, would have better vanquished the enemy. However the powers of opium in analogous cases is of comparative recent discovery, and the battle-field, with all its dreadful privations, is not the place we look for other than surgical achievements. The axiom that "no purgative medicine whatever should be given to a person with a penetrating wound of the abdomen," is strongly and justly insisted upon.

We have perused Mr. Guthrie's work with great pleasure, and strongly recommend it to the notice of our readers, distinguished, as it is, by the same independent spirit of observation, and the ability and originality which characterize his other productions.

